# Dimethyl sulfoxide-free cryopreservation solution containing trehalose, dextran 40, and propylene glycol for therapy with human adipose tissue-derived mesenchymal stromal cells

**DOI:** 10.1007/s10616-022-00541-3

**Published:** 2022-08-12

**Authors:** Yasutaka Fujita, Masuhiro Nishimura, Tamaki Wada, Natsuki Komori, Takeshige Otoi

**Affiliations:** 1 Research and Development Center, Otsuka Pharmaceutical Factory, Inc, Naruto, Tokushima 772-8601 Japan; 2 Bio-Innovation Research Center, Tokushima University, 2272-2 Ishii, Myozai-gun, Tokushima 779-3233 Japan

**Keywords:** Cryopreservation, Mesenchymal stromal cells, Propylene glycol, Trehalose, Dextran

## Abstract

We evaluated a dimethyl sulfoxide (Me2SO)-free cryopreservation solution to freeze human adipose-derived mesenchymal stromal cells (hADSCs). In the first experiment, we compared the combined effects of 3% trehalose (3 T) and 5% dextran (5D) in lactated Ringer’s solution (LR) as a cryopreservation base solution containing 10% propylene glycol (PG). The cell viability of hADSCs immediately after thawing was significantly higher (p < 0.05) in LR supplemented with 3 T (LR-3 T) and with 3 T and 5D (LR-3 T-5D) than in LR. In the second experiment, we compared the cell characteristics of hADSCs freeze-thawed in LR-3 T-5D containing either 10% Me2SO or 10% PG. The cell viability, annexin V-positive ratio, colony-forming capacity, cell proliferation, cell surface antigen positivity, adipogenic differentiation, osteogenic differentiation, and genetic response to cytokine stimulation of hADSCs immediately after thawing were similar between the LR-3 T-5D containing 10% Me2SO and 10% PG. In the third experiment, we examined various concentrations of PG on the cell proliferative capacity of freeze-thawed hADSCs. The cell proliferative capacity of hADSCs frozen with LR-3 T-5D containing 2.5% to 5% PG was significantly higher (p < 0.05) than LR-3 T-5D containing 10% PG. Furthermore, the cell proliferative capacity of hADSCs frozen with LR-3 T-5D containing 4% PG was similar to that of fresh hADSCs. These results indicate that the combination of 3 T-5D in an LR solution as a basic solution is effective for post-thaw cell viability, and that the optimal concentration of PG to maintain the cell characteristics of hADSCs frozen with LR-3 T-5D is 2.5% to 5%, which is promising for cell therapy applications.

## Introduction

Cryopreservation of cells is generally performed using a solution containing about 10% dimethyl sulfoxide (Me2SO). This technique was established in the 1950s (Lovelock and Bishop 1959) and is now commonly used in cell banks. However, side effects associated with the transplantation of stem cells with Me2SO have been reported, including nausea, vomiting, cardiac arrhythmias, neurological symptoms, respiratory arrest, renal/hepatic dysfunction, and allergies (Santos et al. 2003; Windrum et al. 2005; Cox et al. 2012; Shu et al. 2014; Awan et al. 2020). In addition, a portion of the administered Me2SO is reduced to dimethyl sulfide in the body, which is then secreted through the skin and exhaled air, causing a foul, garlic-like odor (Santos et al. 2003; Shu et al. 2014; Awan et al. 2020). Therefore, there is a need for a cryopreservation solution that can reduce the side effects of Me2SO and that has the same level of performance as Me2SO-containing products, assuming that it is administered to humans.

Me2SO stimulates cell differentiation in some types of cells. For example, mouse bone marrow mesenchymal stem cells are differentiated into cardiomyocytes by Me2SO (Young et al. 2004). Cryopreservation of human ES cells in Me2SO-containing cryopreservation solution decreases the expression of Oct-4, an undifferentiated marker (Katkov et al. 2006), and Me2SO affects the epigenetic profile of mouse embryoid bodies (Iwatani et al. 2006). The human promyelocytic leukemia cell line HL-60 is differentiated into granulocytes by Me2SO (Jiang et al. 2006). However, these effects are unfavorable for maintaining cell characteristics during cell cryopreservation. Therefore, given concerns over maintaining stemness in addition to toxicity, a Me2SO-free cryopreservation solution is desired.

We have shown the usefulness of lactated Ringer’s solution supplemented with 3% trehalose and 5% dextran (LR-3 T-5D) with 10% Me2SO as a cryopreservation solution (Fujita et al. 2021). We have also evaluated the procedure of cell washing to remove the effect of Me2SO after thawing the cells (Fujita et al. 2021). However, there are concerns over the effects of Me2SO when cells are thawed and used directly in clinical applications. Therefore, we have compared the effects of the cell membrane penetrating cryoprotectants propylene glycol (PG), ethylene glycol, and glycerol with those of Me2SO, and confirmed that LR-3 T-5D with 10% PG results in a similar cell proliferative capacity as LR-3 T-5D with 10% Me2SO (Fujita et al. 2021). In the present study, we compared the performance of LR-3 T-5D with 10% PG to that of LR-3 T-5D with 10% Me2SO on cell specifications (cell surface antigen positivity, adipogenic differentiation, osteogenic differentiation, and genetic response to cytokine stimulation) of hADSCs.

PG is commonly used as a solvent for intravenous, oral, and topical pharmaceutical preparations and is generally considered safe (Zar et al. 2007). PG is metabolized by the liver to form lactate, acetate, and pyruvate. PG toxicity includes the development of serum hyperosmolality, lactic acidosis, and kidney failure. A maximum dose has not been recommended in the literature for intravenous therapy with PG. If one uses the maximum recommended dose of lorazepam, a PG dose of 2.9 g/h or 69 g/day would be presumably safe in the absence of risk factors. PG is thus a candidate for the substitution of Me2SO for cell therapy if it is used in safe doses. Therefore, in this study, we optimized the concentration of PG for cryopreservation of hADSCs.

## Materials and methods

### Study design

The present study was approved by the ethics committee of Otsuka Pharmaceutical Factory, Inc.

We performed three experiments. First, the effects as a cryoprotective vehicle with 10% PG were compared among LR, LR-3 T, and LR-3 T-5D. Second, the cell characteristics (viability, annexin V-positive ratio, colony-forming capacity, differentiation ability, cell surface markers, and mRNA expression level) after cryopreservation in liquid nitrogen were compared between LR-3 T-5D with Me2SO and LT-3 T-5D with PG. Third, the concentration of PG was optimized.

### Components of the solutions

Lactated Ringer’s solution (LR; Lactec® Injection), lactated Ringer’s solution with 3% trehalose (LR-3 T; Cellstor-W), and lactated Ringer’s solution with 3% trehalose and 5% dextran 40 (LR-3 T-5D; Cellstor-S) were supplied by Otsuka Pharmaceutical Factory, Inc. (Tokushima, Japan). Me2SO (CultureSure®DMSO) was purchased from Fujifilm Wako Pure Chemical Co. (Osaka, Japan). PG was purchased from Maruishi Pharmaceutical. Co., Ltd. (Osaka, Japan) and Fujifilm Wako Pure Chemical Co. (Osaka, Japan). The base solution (LR, LR-3 T, or LR-3 T-5D) with 4 or 10% cryoprotectant was prepared by mixing base solution and cryoprotectant at ratios of 24:1 or 9:1 (vol/vol), respectively. The compositions of LR-3 T, LR-3 T-5D, LR-3 T-5D with 4 or 10% Me2SO, and LR-3 T-5D with 4 or 10% PG are described in Table 1.

**Table 1:** Compositions of preservation solutions (LR-3 T and LR-3 T-5D) and the cryopreservation solution (LR-3 T-5D with 10% Me2SO and LR-3 T-5D with 10% PG)

Components	LR-3 T	LR-3 T-5D	LR-3 T-5D with 4% Me2SO	LR-3 T-5D with 10% Me2SO	LR-3 T-5D with 4% PG	LR-3 T-5D with 10% PG
Na^+^	130 mEq/L	130 mEq/L	1.25 mEq/L	117 mEq/L	1.25 mEq/L	117 mEq/L
K^+^	4 mEq/L	4 mEq/L	3.8 mEq/L	3.6 mEq/L	3.8 mEq/L	3.6 mEq/L
Ca^2+^	3 mEq/L	3 mEq/L	2.9 mEq/L	2.7 mEq/L	2.9 mEq/L	2.7 mEq/L
Cl^−^	109 mEq/L	109 mEq/L	105 mEq/L	98 mEq/L	105 mEq/L	98 mEq/L
Lactate^−^	28 mEq/L	28 mEq/L	27 mEq/L	25 mEq/L	27 mEq/L	25 mEq/L
Trehalose	3%	3%	2.88%	2.7%	2.88%	2.7%
Dextran40	–	5%	4.8%	4.5%	4.8%	4.5%
DMSO	–	–	4%	10%	–	–
PG	–	–	–	–	4%	10%

*LR-3 T* lactated Ringer’s solution with 3% trehalose, *LR-3 T-5D* lactated Ringer’s solution with 3% trehalose and 5% dextran 40, *LR-3 T-5D with 4% Me2SO* LR-3 T-5D with 4% dimethyl sulfoxide, *LR-3 T-5D with 10% Me2SO* LR-3 T-5D with 10% dimethyl sulfoxide, *LR-3 T-5D with 4% PG* LR-3 T-5D with 4% propylene glycol, *LR-3 T-5D with 10% PG* LR-3 T-5D with 10% propylene glycol

### Preparation of hADSCs

Human ADSCs (Female, 44Y or 32Y, PT5006, Lot No. 0000692059 or 19TL200176; Lonza Walkersville, Inc., Walkersville, MD, USA) were used in this study; 44Y, and 32Y indicate the ages of the donors. Human ADSCs were seeded in a 75 cm^2^ flask with 15 mL of medium prepared from a medium kit (PT-4505 ADSC BulletKit™, Lonza Walkersville, Inc.) and maintained at 37 °C in a humidified atmosphere of 5% CO_2_. All of the medium was changed every 3 or 4 days. Cells were passaged at approximately 90% confluency, and passages 2, 3, or 5 (3, 4, or 6 after cell preparation) were used for the experiments. Cells were collected as described in previous report (Fujita et al. 2021).

### Cryopreservation of hADSCs

The hADSCs suspended in LR-3 T were transferred to a 15- or 50 mL conical tube, centrifuged (210×*g*, 5 min, at room temperature), and the supernatant was aspirated. hADSCs were resuspended with LR, LR-3 T, or LR-3 T-5D containing 10% Me2SO or 10% PG so that the final cell concentration was 1.0 to 3.0 × 10^6^ cells/mL. One milliliter of suspension was dispensed into each cryovial (Nunc™ CryoTube™ Vials, size 1.8 mL, Thermo Fisher Scientific Inc., Waltham, MA, USA). Immediately after dispensing, the vial was placed in a BICELL (Nihon Freezer Co., Ltd., Tokyo, Japan) and stored at − 80 °C for approximately 24 h. Then the vial was transferred from the BICELL or the freeze box to a freeze box preincubated in liquid nitrogen and stored in liquid nitrogen for 46 days at the longest. The length of the storage period in each experiment was different among experiments and described in figure legends.

### Thawing of cryopreservation solution

The frozen vials in which the hADSCs were stored were quickly thawed in a thermostat bath heated to 37 °C. After thawing, they were used for the following studies.

### Cell viability and viable cell recovery ratio

The total numbers of cells and dead cells were counted manually with a plastic cell counting plate (OneCell Counter, Bio Medical Science, Ltd., Tokyo, Japan) after trypan blue staining. Cell viability and viable cell recovery ratio were calculated according to the formulae below.

$${\text{Cell viability }}\left[ \% \right] = \left( {{\text{total number of cells }} - {\text{ number of dead cells}}} \right) / \left( {\text{total number of cells}} \right) \times 100$$

$${\text{Viable cell recovery ratio }}\left[ \% \right] = {\text{number of viable cells in 1 mL at each time point }}/{\text{ number of viable cells in 1 mL before freezing }} \times 100$$

### Annexin V staining

Suspended cells were stained using an Annexin V-FITC Kit (Beckman Coulter, Brea, CA, USA). Measurements were performed using a Gallios flow cytometer (Beckman Coulter, Indianapolis, IN, USA).

### Colony-forming capacity

Colony-forming capacity was evaluated as described in previous report (Fujita et al. 2021). Briefly, Cells were plated at a density of 15 cells/cm^2^ (315 cells in a 21 cm^2^ culture dish). After 8 days, the cells were stained with Giemsa. Colonies of more than 50 cells were counted. The colony-forming efficiency of cells was calculated by dividing the number of colonies per dish by the number of cells (315) seeded per dish.

### Cell proliferation curve

Colony-forming capacity was evaluated as described in previous report (Fujita et al. 2021). Briefly, hADSCs were seeded at a density of 5.6 × 10^3^ cells/cm^2^ (5.0 × 10^4^ cells/well). The total number of cells was counted with a OneCell Counter on 1, 3, 5, and 7 days after seeding.

### Adipogenic and osteogenic differentiation ability

Adipogenic differentiation was induced according to the Poietics™ human ADSCs adipogenesis protocol (Lonza Walkersville, Inc.) and evaluated by Oil Red O staining. Osteogenic differentiation was induced according to the Poietics™ human ADSCs osteogenesis protocol (Lonza Walkersville, Inc.) and evaluated with an alkaline phosphatase staining kit (AK20, Cosmo Bio Co., Ltd., Tokyo, Japan) and a calcified nodule staining kit (AK21, Cosmo Bio Co., Ltd.).

### Cell surface markers

To examine the surface immunophenotypes of the cells, 2 × 10^5^ cells in 20 µL of staining buffer with fetal bovine serum (BD Biosciences, San Jose, CA, USA) were incubated for 60–120 min on ice with phycoerythrin-labeled antibodies against human CD14, CD29, CD31, CD34, CD44, CD45, CD73, CD90, CD105, and HLA-DR (BD Biosciences) or the respective isotype controls (BD Biosciences). After washing, the labeled cells were analyzed using a Gallios flow cytometer (Beckman Coulter).

### mRNA expression level after cytokine stimulation

The changes in mRNA expression of the following human genes (Table 2) were examined in hADSCs under the following conditions with subcultures both before and after frozen storage. Either freshly prepared or thawed hADSC suspensions (1 × 10^6^ cells) were diluted with culture medium (1:9) and were centrifuged at 800×*g* for 5 min. Each cell pellet was diluted in medium and divided in half (5 × 10^5^ cells each). hADSCs were cultured in medium with or without both 5 ng/mL recombinant human interferon gamma (IFN-γ; R&D Systems, Inc., Minneapolis, MN, USA) and 5 ng/mL recombinant human tumor necrosis factor alpha (TNF-α; R&D Systems, Inc.) using 25 cm^2^ culture flasks for 24 h. Then, hADSCs were collected with trypsinization and were lysed in RLT buffer (Qiagen Inc., Germantown, MD, USA) with 2-mercaptoethanol. Total RNA was isolated using RNeasy columns (Qiagen Inc.) following the manufacturer’s instructions, and the RNA amount was measured by a NanoDrop 200c (Thermo Fisher Scientific Inc.). cDNA was synthesized from 1 µg of RNA using a High Capacity cDNA Reverse Transcription Kit with RNase Inhibitor (Thermo Fisher Scientific Inc.) with the following conditions: 25 °C for 10 min, 37 °C for 120 min, and 85 °C for 5 min, and was stored at − 30 °C. 25 ng of total RNA equivalent cDNA per reaction was mixed with TaqMan Fast Advanced Master Mix (Thermo Fisher Scientific Inc.) (1:1) into a TaqMan Array Plate 32 Plus (Fast, 0.1 mL) (Thermo Fisher Scientific Inc.). PCR assay was performed using the Applied Biosystems 7500 Fast Real-Time PCR System with the following profile: 1 cycle at 50 °C for 2 min, 1 cycle at 95 °C for 20 s, and 40 cycles at 95 °C for 3 s and 60 °C for 30 s. The threshold cycle (Ct) was calculated by the instrument's software (7500 Fast System ver. 2.3). The relative expression of each mRNA was calculated using the ΔCt method (where ΔCt is the value obtained by subtracting the Ct value of hypoxanthine phosphoribosyltransferase 1 (HPRT1) mRNA from the Ct value of the target mRNA). Specifically, 2^−(ΔCt)^ is expressed as the amount of target mRNA relative to HPRT1 mRNA. Target genes were selected from immunomodulatory genes, indoleamine 2,3-dioxygenase 1 (IDO1), hepatocyte growth factor (HGF), prostaglandin E synthase (PTGES), prostaglandin-endoperoxide synthase 2 (PTGS2, also known as cyclooxygenase 2, COX2), programmed death 1 ligand-1 (PD-L1), and chemokine (C–C motif) ligand 5 (CCL5, also known as regulated on activation, normal T cell expressed and secreted, RANTES).

**Table 2:** Real-time PCR primers

Gene Symbols	Assay ID
HPRT1	Hs99999909_m1
CCL5 (RANTES)	Hs99999048_m1
PD-L1 (CD274)	Hs00204257_m1
HGF	Hs00300159_m1
IDO1	Hs00984148_m1
PTGES	Hs00610420_m1
PTGS2 (COX2)	Hs00153133_m1

Assay IDs of the primers used for TaqMan-based PCR (Thermo Fisher Scientific Inc.) are indicated with gene symbols.

*HPRT1* hypoxanthine phosphoribosyltransferase 1 (housekeeping gene), *CCL5* chemokine (C–C motif) ligand 5 (RANTES, regulated on activation, normal T cell expressed and secreted), *PD-L1* programmed death 1 ligand-1, *HGF* hepatocyte growth factor, *IDO1* indoleamine 2,3-dioxygenase 1, *PTGES* prostaglandin E synthase, *PTGS2* prostaglandin-endoperoxide synthase 2, *COX2* cyclooxygenase 2

### Analysis and statistics

Results are presented as the mean ± standard deviation (SD). Statistical analysis was performed using Dunnett’s multiple comparison test, Tukey’s multiple comparison test, and Student’s *t* test with a significance level of p < 0.05. Data were analyzed with SAS 9.4 (SAS Institute Inc., Cary, NC, USA).

## Results

### Comparison of LR, LR-3 T, and LR-3 T-5D as cryoprotective vehicle solutions with 10% PG

**Fig. 1:**
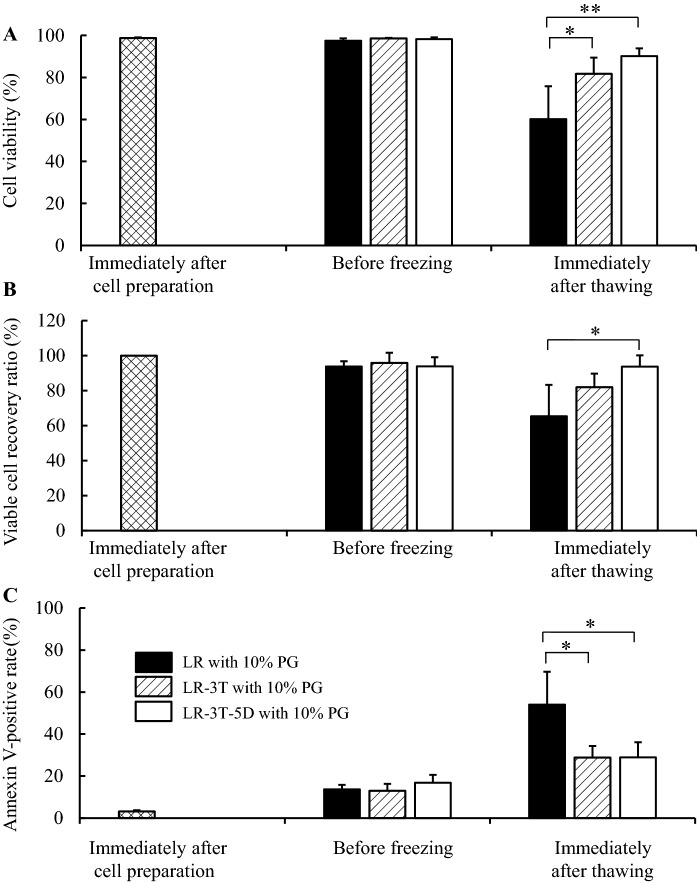
Cell viability (**A**), viable cell recovery ratio (**B**), and annexin V-positive ratio (**C**) of hADSCs cryopreserved using LR with 10% PG, LR-3 T with 10% PG, or LR-3 T-5D with 10% PG. hADSCs at passage three were suspended at a density of 1.0 × 10^6^ cells/mL using LR with 10% PG, LR-3 T with 10% PG, or LR-3 T-5D with 10% PG. Vials containing 1 mL of the suspension were put into a BICELL and frozen in a freezer at − 80 °C for a day. Then, the vials were transferred from the BICELL to a freeze box preincubated in liquid nitrogen then stored in liquid nitrogen for 36 or 37 days. The data are presented as the mean ± SD (n = 4). Statistical analysis was performed using Tukey’s test. *p < 0.05, **p < 0.01

The effects as cryoprotective vehicle solutions with 10% PG were compared among LR, LR-3 T, and LR-3 T-5D (Fig. 1). hADSCs were suspended in LR with 10% PG, LR-3 T with 10% PG, or LR-3 T-5D with 10% PG, then cryopreserved for more than 1 month. There was no significant difference in cell viability, viable cell recovery ratio, or annexin V-positive ratio of hADSCs before freezing among LR with 10% PG, LR-3 T with 10% PG, and LR-3 T-5D with 10% PG. Cell viability immediately after thawing was significantly higher (p < 0.05) in LR-3 T with 10% PG and LR-3 T-5D with 10% PG than in LR with 10% PG (Fig. 1A). The viable cell recovery ratio immediately after thawing was significantly higher (p < 0.05) in LR-3 T-5D with 10% PG than in LR with 10% PG (Fig. 1B). The annexin V-positive ratio immediately after thawing was significantly lower (p < 0.05) in LR-3 T with 10% PG and LR-3 T-5D with 10% PG than in LR with 10% PG (Fig. 1C).

### Cell characteristics

**Fig. 2:**
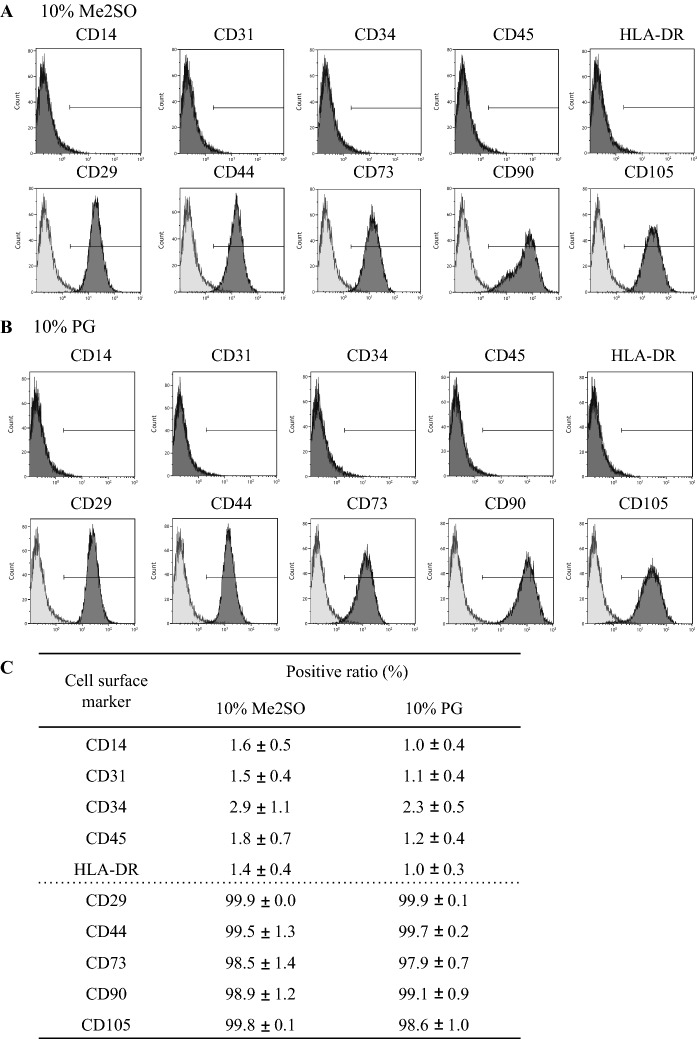
Representative charts of flow cytometry analyses of cell surface markers of hADSCs cryopreserved using LR-3 T-5D with 10% Me2SO (**A**) and LR-3 T-5D with 10% PG (**B**). Positive ratios of cell surface markers are presented as the mean ± SD (n = 3) (**C**). hADSCs at passage 6 were suspended at a density of 3.0 × 10^6^ cells/mL using LR-3 T-5D with 10% Me2SO or LR-3 T-5D with 10% PG. Vials containing 1 mL of the suspension were put into a BICELL and frozen in a freezer at − 80 °C for a day. Then, the vials were transferred from the BICELL to a freeze box preincubated in liquid nitrogen and stored in liquid nitrogen for 18, 44, or 46 days. Isotype controls and antigen stains are represented as the light and dark shaded areas, respectively

CD14, CD31, CD34, CD45, and HLA-DR in cells with LR-3 T-5D containing 10% PG were negative, as were those in cells with LR-3 T-5D with 10% Me2SO. CD29, CD44, CD73, CD90, and CD105 in cells with LR-3 T-5D containing 10% PG were positive, as were those in cells with LR-3 T-5D with 10% Me2SO (Fig. 2).

**Fig. 3:**
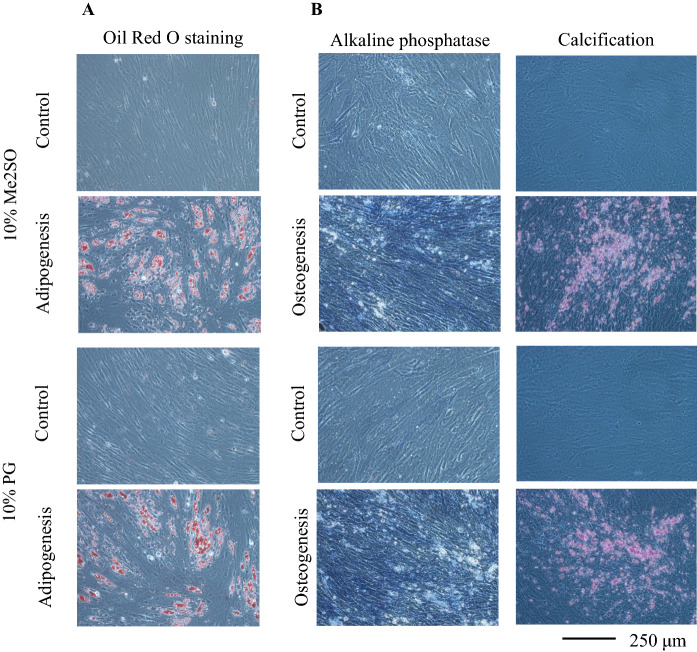
Representative images after adipogenesis differentiation (**A**) and osteogenesis differentiation (**B**) of hADSCs seeded immediately after thawing following cryopreservation for 46 days. hADSCs at passage four were suspended at a density of 1.0 × 10^6^ cells/mL using LR-3 T-5D with 10% Me2SO or LR-3 T-5D with 10% PG. Vials containing 1 mL of the suspension were put into a BICELL and frozen in a freezer at − 80 °C for a day. Then, the vials were transferred from the BICELL to a freeze box preincubated in liquid nitrogen and stored in liquid nitrogen for 46 days. The cells immediately after thawing were seeded and cultured in normal culture medium (control), medium for adipogenic differentiation, or medium for osteogenic differentiation. Adipogenic and osteogenic differentiated cells were cultured for 9 and 17 days, respectively. Adipogenic differentiation was confirmed by Oil Red O staining. Osteogenic differentiation was confirmed by alkaline phosphatase staining and calcified nodule staining

Adipogenic differentiation and osteogenic differentiation were induced using hADSCs immediately after thawing following cryopreservation with LR-3 T-5D containing 10% Me2SO or 10% PG (Fig. 3A, B respectively). Adipocytes containing oil droplets stained with Oil Red O were observed both in 10% Me2SO and 10% PG groups, but not in control cultures without the induction of adipogenic differentiation. Similarly, osteoblasts confirmed by alkaline phosphatase staining and calcification staining were observed both in 10% Me2SO and 10% PG groups, but not in control cultures without the induction of osteogenic differentiation.

**Fig. 4:**
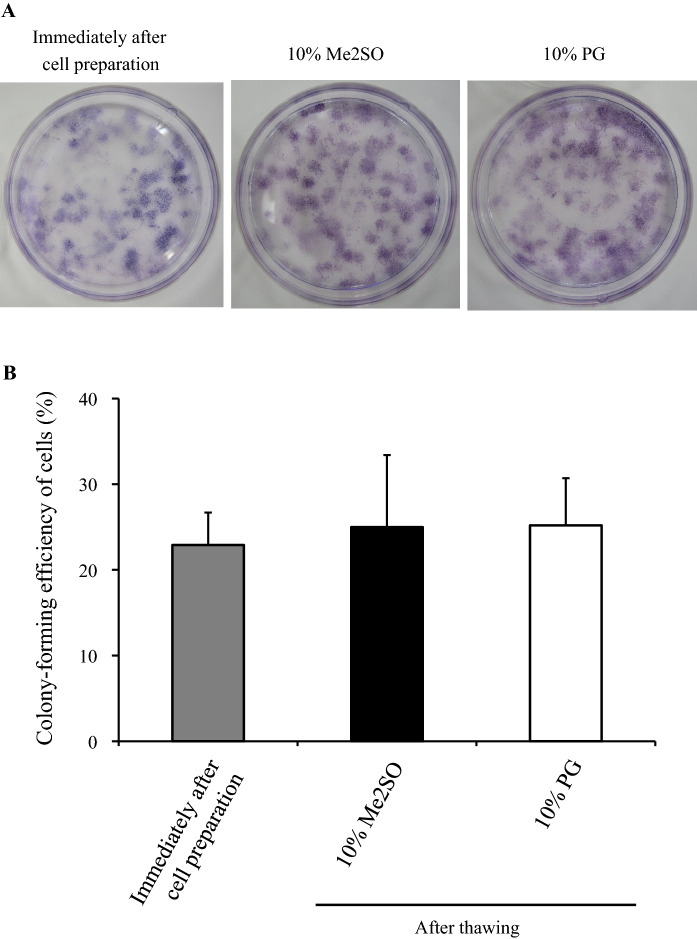
Colony-forming capacity of hADSCs immediately after thawing. hADSCs at passage four were suspended at a density of 1.0 × 10^6^ cells/mL in LR-3 T-5D with 10% Me2SO or 10% PG. Vials containing 1 mL of the suspension were put into a BICELL and frozen in a freezer at − 80 °C for a day. Then, these vials were transferred from the BICELL to a freeze box preincubated in liquid nitrogen and stored in liquid nitrogen for 31 days. The data are presented as the mean ± SD (n = 4). The values ‘after cell preparation’ were those at the time of suspension in LR-3 T during cell preparation. Statistical analysis was performed using Student’s t test, and there was no significant difference between storage in 10% Me2SO and in 10% PG

Figure 4 shows the colony-forming capacity of hADSCs immediately after thawing following cryopreservation using LR-3 T-5D containing 10% Me2SO or 10% PG. There was no significant difference in colony formation capacity between LR-3 T-5D with 10% Me2SO and LR-3 T-5D with 10% PG.

### mRNA expression level

**Fig. 5:**
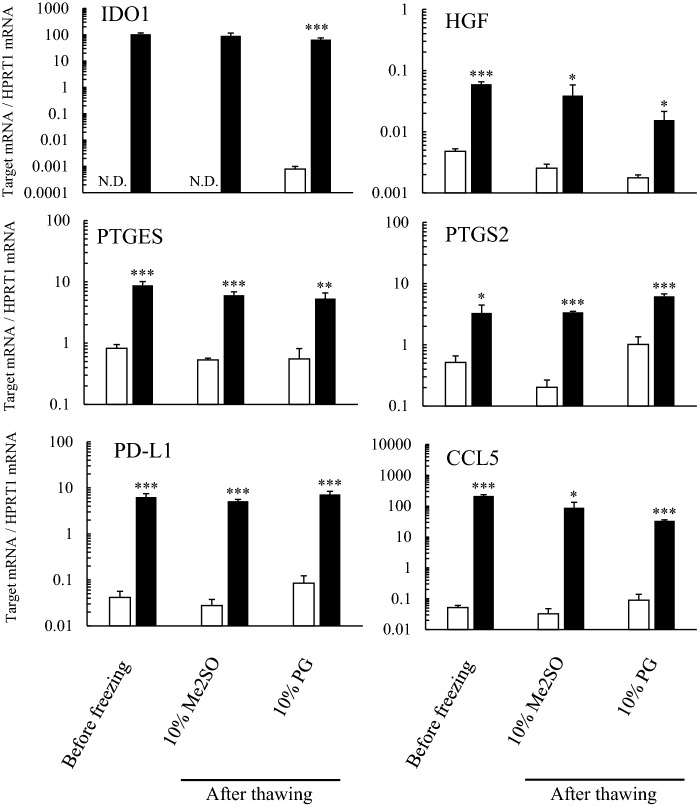
mRNA expression of hADSCs subcultured with cytokine stimulation after thawing. hADSCs at passage four were suspended at a density of 1.0 × 10^6^ cells/mL in LR-3 T-5D with 10% Me2SO or 10% PG. Vials containing 1 mL of the suspension were put into a BICELL and frozen in a freezer at − 80 °C for a day. Then, these vials were transferred from the BICELL to a freeze box preincubated in liquid nitrogen and stored in liquid nitrogen for 5 days. ADSCs before freezing or after thawing were cultured in medium with or without both 5 ng/mL recombinant human interferon gamma and 5 ng/mL recombinant human tumor necrosis factor alpha for 24 h. The open and closed columns are without and with cytokine stimulation, respectively. HPRT1 hypoxanthine phosphoribosyltransferase 1 (housekeeping gene), CCL5 chemokine (C–C motif) ligand 5, RANTES regulated on activation, normal T cell expressed and secreted, PD-L1 programmed death 1 ligand-1, HGF hepatocyte growth factor, IDO1 indoleamine 2,3-dioxygenase 1, PTGES prostaglandin E synthase, PTGS2 prostaglandin-endoperoxide synthase 2, COX2 cyclooxygenase 2. Data are expressed as the ratio of the target mRNA to HPRT1 mRNA. The data are presented as the mean ± SD (n = 3). Statistical analysis was performed using Student’s *t* test between subcultures with and without cytokine stimulation. *p < 0.05, **p < 0.01, ***p < 0.001

Mesenchymal stromal cells respond to inflammatory cytokines and show broad anti-inflammatory and immunomodulatory properties via the secretion of various factors (Pittenger et al. 2019). Thus, we focused on the responses of representative genes such as immunomodulatory genes (IDO-1, PTGES, PTGES2, and CCL5) or a pro-angiogenesis and anti-apoptotic gene (HGF) to cytokine stimulation following cryopreservation of hADSCs. To confirm the cytokine response of hADSCs, fresh cells (immediately after cell preparation) or cells after thawing following cryopreservation were cultured with or without 5 ng/mL recombinant human IFN-γ and 5 ng/mL recombinant human TNF-α for 24 h. All gene expressions were markedly and significantly higher (p < 0.05) in subcultures with cytokines in hADSCs immediately after cell preparation or after thawing following cryopreservation both in LR-3 T-5D with 10% Me2SO and LR-3 T-5D with 10% PG groups than in subcultures without cytokines (Fig. 5).

### Optimization of the concentration of PG

**Fig. 6:**
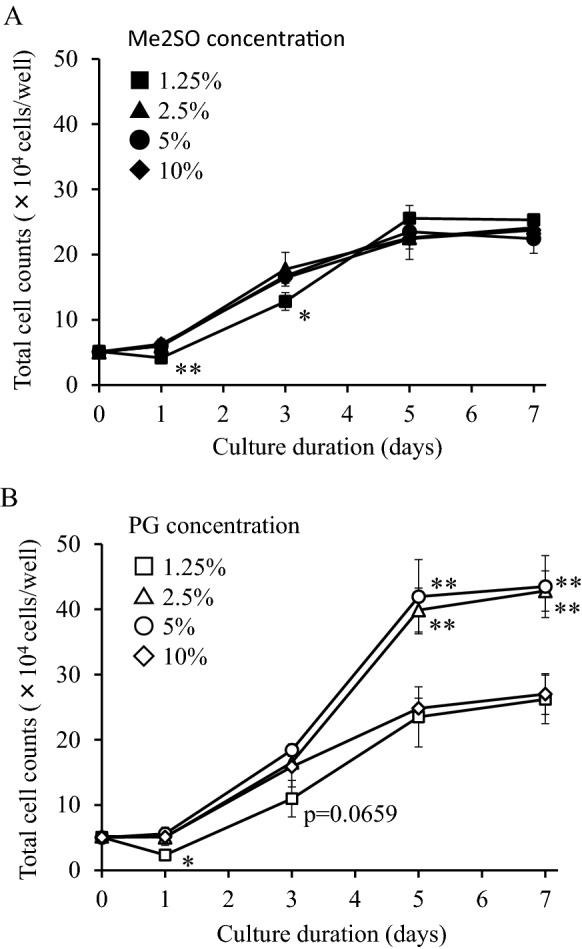
Proliferation curve of hADSCs seeded immediately after thawing that were cryopreserved using LR-3 T-5D containing various concentrations of Me2SO (**A**) or PG (**B**). hADSCs at passage 4 were suspended at a density of 1.0 × 10^6^ cells/mL using LR-3 T-5D with 1.25 to 10% Me2SO or LR-3 T-5D with 1.25 to 10% PG. Vials containing 1 mL of the suspension were put into a BICELL and frozen in a freezer at − 80 °C for a day. Then, the vials were transferred from the BICELL to a freeze box preincubated in liquid nitrogen and stored in liquid nitrogen for 31 days. Immediately after thawing, the cells were seeded at a density of 5.6 × 10^3^ cells/cm^2^ (5 × 10^4^ cells/well) in a 6-well plate. The data are presented as the mean ± SD (n = 3). Statistical analysis was performed using Dunnett's test. *p < 0.05, **p < 0.01 vs. closed or open diamonds

Figure 6 shows the cell proliferation curve of hADSCs during subcultures seeded immediately after thawing of hADSCs cryopreserved using LR-3 T-5D containing various concentrations of Me2SO or PG. The proliferation of the LR-3 T-5D containing 1.25% Me2SO group was slower than that of the LR-3 T-5D containing 10% Me2SO group up to 3 days after seeding, while the LR-3 T-5D containing 2.5% or 5% Me2SO groups showed the same levels of proliferation as that of the LR-3 T-5D containing 10% Me2SO group (Fig. 6A). However, the total cell counts at 5 and 7 days after seeding of the LR-3 T-5D containing 2.5% or 5% PG groups were significantly higher (p < 0.05) than that of the LR-3 T-5D containing 10% PG group (Fig. 6B).

**Fig. 7:**
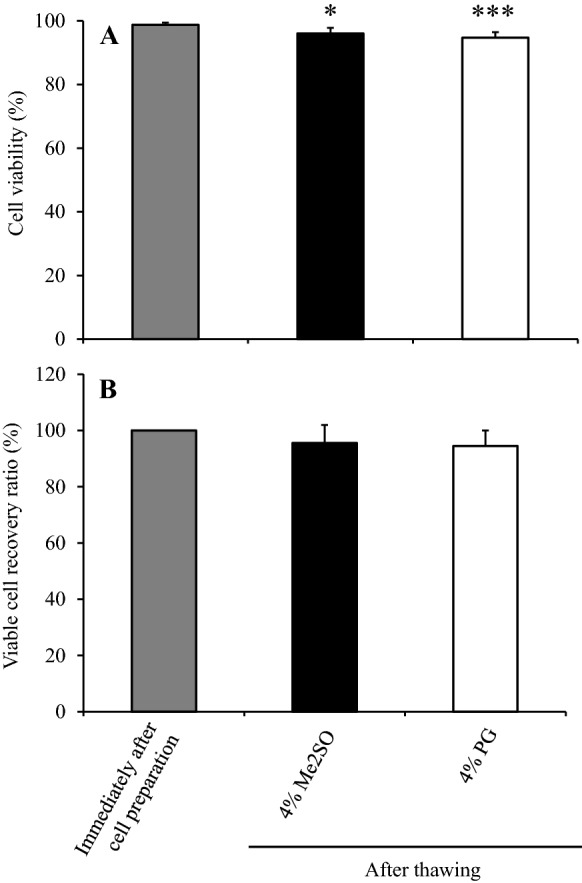
Cell viability (**A**) and viable cell recovery ratio (**B**) of hADSCs immediately after thawing of samples cryopreserved in 4% Me2SO or 4% PG. Test solutions containing Me2SO or PG were prepared by adding Me2SO or PG to LR-3 T-5D. hADSCs at passage five were suspended at a density of 1.0 × 10^6^ cells/mL using test solutions containing Me2SO or PG. For the data before freezing, hADSCs suspended in LR-3 T-5D were used. Vials containing 1 mL of the suspension were put into a BICELL and frozen in a freezer at − 80 °C for 3 ~ 4.5 h. Then, the vials were transferred from the BICELL to a freeze box preincubated in liquid nitrogen and stored in liquid nitrogen for 23 days. The data are presented as the mean ± SD (n = 6). Statistical analysis was performed using Dunnett's test. *p < 0.05, ***p < 0.001 vs. before freezing

We compared cell viability, viable cell recovery ratio, and the cell proliferation curve between before freezing and immediately after thawing in hADSCs cryopreserved using LR-3 T-5D containing 4% (the representative concentration for 2.5% to 5%) Me2SO or PG. Cell viability immediately after thawing was approximately 95%, but significantly lower (p < 0.05) in hADSCs cryopreserved using LR-3 T-5D containing 4% Me2SO or 4% PG than before freezing (98.8%) (Fig. 7A). The viable cell recovery ratio in hADSCs cryopreserved using LR-3 T-5D containing 4% Me2SO or PG was also about 95% (Fig. 7B).

**Fig. 8:**
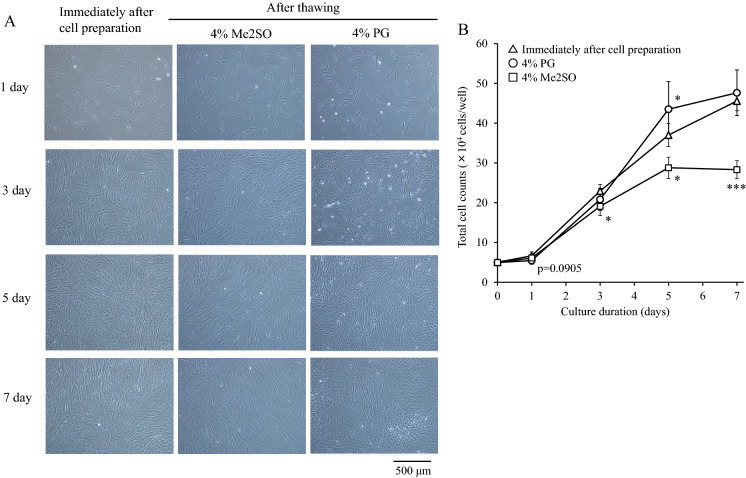
Photographs over time (**A**) and proliferation curve (**B**) of hADSCs seeded immediately after thawing of samples cryopreserved in 4% Me2SO or 4% PG. Test solutions containing Me2SO or PG were prepared by adding Me2SO or PG to LR-3 T-5D. hADSCs at passage five were suspended at a density of 1.0 × 10^6^ cells/mL using test solutions containing Me2SO or PG. For the data before freezing, hADSCs suspended in LR-3 T-5D were used. Vials containing 1 mL of the suspension were put into a BICELL and frozen in a freezer at − 80 °C for 3 ~ 4.5 h. Then, the vials were transferred from the BICELL to a freeze box preincubated in liquid nitrogen and stored in liquid nitrogen for 23 days. Immediately after thawing, the cells were seeded at a density of 5.6 × 10^3^ cells/cm^2^ (5 × 10^4^ cells/well) in a 6-well plate. The data are presented as the mean ± SD (n = 6). Statistical analysis was performed using Dunnett's test. *p < 0.05, ***p < 0.001 vs. before freezing

As shown in the photographs (Fig. 8A), the hADSCs at 5 days after seeding before freezing and those frozen in LR-3 T-5D containing 4% Me2SO or 4% PG reached 100% confluence. The total cell count of LR-3 T-5D containing 4% PG was comparable to that before freezing, while that of LR-3 T-5D containing 4% Me2SO was significantly lower (p < 0.05) than that before freezing at 3 to 7 days after seeding (Fig. 8B).

## Discussion

There is concern about side effects of Me2SO administration. Therefore, it is desirable to wash the cells and replace the solution after thawing to avoid Me2SO exposure. However, taking this approach prior to clinical use is cumbersome. In contrast, a Me2SO-free cryopreservation solution with 10% PG has the advantage of avoiding Me2SO-related side effects without such procedures.

We have already confirmed the additive effects of trehalose and dextran 40 added to LR for refrigerated storage (Fujita et al. 2020), room temperature storage (Fujita et al. 2020), and cryopreservation with 10% Me2SO (Fujita et al. 2021). In this study, similarly, we found that cell viability immediately after thawing was improved by LR-3 T with 10% PG and LR-3 T-5D with 10% PG compared with LR with 10% PG. The viable cell recovery ratio immediately after thawing also increased in LR-3 T-5D with 10% PG than in LR with 10% PG. Moreover, the annexin V-positive ratio immediately after thawing decreased in LR-3 T with 10% PG and LR-3 T-5D with 10% PG than in LR with 10% PG. These data indicated the additive effects of trehalose and dextran 40 for cryopreservation with 10% PG. Therefore, we concluded that the presence of both trehalose and dextran 40 is essential to keep the higher quality of hADSCs during the freeze–thaw process with propylene glycol.

CD14, CD31, CD45, and HLA-DR are negative markers of hADSCs (Bourin et al. 2013; Di Battista et al. 2014). CD14 is prominently expressed on monocytes and macrophages (Dominici et al. 2006; Di Battista et al. 2014), and CD-31 is a platelet endothelial cell adhesion molecule (PECAM-1) that is found on the surface of platelets, leukocytes, and endothelial cells (Bourin et al. 2013). CD45 is a common leukocyte antigen (Dominici et al. 2006; Bourin et al. 2013; Di Battista et al. 2014), and HLA-DR is a HLA class II cell surface receptor (Dominici et al. 2006; Di Battista et al. 2014). CD34 has been both reported as a negative marker (Di Battista et al. 2014) and a positive marker (Bouri et al. 2013) of hADSCs. The percentage of CD34-positive hADSCs depends on the class of CD34 antibody, the method of adipose tissue harvest, the degree of vascular hemorrhage, the subsequent digestion and isolation techniques, the culture conditions, and passage number (Bouri et al. 2013). In this study, CD34 was used as a negative marker. CD34 marks primitive hematopoietic progenitors and endothelial cells (Dominici et al. 2006; Bourin et al. 2013; Di Battista et al. 2014). CD29, CD44, CD73, CD90, and CD105 are positive markers of hADSCs (Bouri et al. 2013; Di Battista et al. 2014). These negative and positive cell surface markers remained negative and positive respectively after thawing following cryopreservation in LR-3 T-5D with 10% PG, indicating that the cell characteristics of hADSCs were preserved during the freeze–thaw process.

One criterion for defining MSCs is multipotent differentiation potential (Dominici et al. 2006; Bouri et al. 2013; Di Battista et al. 2014). We confirmed that hADSCs still had adipogenic and osteogenic differentiation abilities after thawing following cryopreservation in LR-3 T-5D with 10% PG. This result indicated that the freeze-thaw process did not affect the differentiation ability of hADSCs. There were no obvious differences in colony-forming capacity before and after cryopreservation using LR-3 T-5D with 10% PG. This result also indicated that the cryopreservation did not affect the ratio of cells with proliferation ability.

MSCs respond to inflammatory cytokines and show broad anti-inflammatory and immunomodulatory properties via the secretion of various factors (Krampera et al. 2013; Pittenger et al. 2019). The International society for cellular therapy (ISCT) recommends that a standard immune plasticity assay should use IFN-γ ± TNF-α as a model in vitro priming agent (Krampera et al. 2013). Thus, we used culture media containing IFN-γ and TNF-α as an immune-stimulation model for hADSCs and evaluated the expression of representative genes IDO1, HGF, PTGES, PTGS2, PD-L1, and CCL5, which are upregulated in cytokine-exposed MSCs (Krampera et al. 2013; Galipeau et al. 2016; Chinnadurai et al. 2018; Guan et al. 2018). These genes have various function related to efficacy of MSCs. For example, IDO1 catalyzes the rate-limiting step of tryptophan catabolism along the degradation pathway, leading to tryptophan starvation and production of tryptophan metabolites kynurenines, resulting in inhibiting effector T-cell responses and promoting Treg development (Guan et al. 2018). HGF is antiapoptotic and angiogenic factor (Merimi et al. 2021). Prostaglandin E2 is synthesized by PTGES and PTGS2 (also known as COX2, cyclooxygenase 2). Prostaglandin E2 secreted from bone marrow stromal cells reprograms the macrophages to increase their interleukin-10 production (Németh et al. 2009). Via the interaction of PD-L1 with its receptor PD-1 expressed on T lymphocytes, MSCs suppress the activation of CD4 + T cells, down-regulate interleukin-2 secretion and induce irreversible hyporesponsiveness and cell death (Davies et al. 2017). CCL5 (also known as RANTES, regulated on activation, normal T cell expressed and secreted) recruits T cells to the vicinity of MSCs (Can and Coskun 2020). We confirmed that hADSCs still had a gene expression response to cytokines after thawing following cryopreservation in LR-3 T-5D with 10% PG, the same as that before cryopreservation. This observation suggested that the freeze–thaw processes did not affect the putative effects of hADSCs. In summary, the cell surface markers, adipogenesis and osteogenesis differentiation potency, cell proliferation ability, and gene expression response to cytokine stimulation of hADSCs after thawing following cryopreservation in LR-3 T-5D with 10% PG were comparable to those before cryopreservation and in LR-3 T-5D with 10% Me2SO.

Furthermore, we optimized the concentration of PG for cryopreservation of hADSCs. Interestingly, the total cell counts at 5 days and 7 days after subculture of the 2.5% and 5% PG groups were significantly higher than those of the 10% PG group. However, such a response to concentration was not observed for Me2SO. When we chose 4% as a representative concentration for 2.5–5%, the total cell counts at 5 days and 7 days after subculture of the 4% PG group were comparable to those before freezing and significantly higher than those of the 4% Me2SO group. This suggests that the morphology of hADSCs was changed and cell proliferation was mechanically suppressed by 4% Me2SO, but not by 4% PG. Therefore, our data suggest that the optimal concentration of PG to maintain the cell characteristics of hADSCs frozen with LR-3 T-5D is 2.5–5%.

There are several commercial Me2SO-free cryoprotectants (Awan et al. 2020), but their exact compositions have not been disclosed. The advantage of our solutions is that their exact compositions are disclosed so that users may account for their safety (Table 1). Moreover, all components of our solutions can be prepared from raw materials that are used in various approved drugs. We also confirmed that the optimal concentration of PG to keep the viability of freeze-thawed CD4 positive T cells and CD8 positive T cells was 3–5 percent (unpublished data). It suggested that LR-3 T-5D containing 3–5% PG is possibly effective for other cells.

## Conclusion

The hADSCs cryopreserved in LR-3 T-5D containing 2.5–5% PG were found to maintain their same performance from before freezing. Our results suggest that cryopreservation with PG-containing LR-3 T-5D may maintain the quality of cells for cell therapy. For clinical applications, further in vivo studies on efficacy and safety in combination with each therapeutic cell type are necessary.

## Data Availability

The data that support the findings of this study are available from Otsuka Pharmaceutical Factory, Inc., but restrictions apply to the availability of these data, which were used under licence for the current study, and so are not publicly available. Data are however available from the corresponding author upon reasonable request and with permission of Otsuka Pharmaceutical Factory, Inc.
